# Design and Validation of a Low-Cost Portable Device to Quantify Postural Stability †

**DOI:** 10.3390/s17030619

**Published:** 2017-03-18

**Authors:** Yong Zhu

**Affiliations:** Department of Mechanical Engineering, Wilkes University, Wilkes-Barre, PA 18766, USA; yong.zhu@wilkes.edu; Tel.: +1-570-408-6034

**Keywords:** human balance, postural stability, approximate entropy (ApEn), center-of-pressure (COP), portable device, low-cost assessment device

## Abstract

Measurement of the displacement of the center-of-pressure (COP) is an important tool used in biomechanics to assess postural stability and human balance. The goal of this research was to design and validate a low-cost portable device that can offer a quick indication of the state of postural stability and human balance related conditions. Approximate entropy (ApEn) values reflecting the amount of irregularity hiding in COP oscillations were used to calculate the index. The prototype adopted a portable design using the measurements of the load cells located at the four corners of a low-cost force platform. The test subject was asked to stand on the device in a quiet, normal, upright stance for 30 s with eyes open and subsequently for 30 s with eyes closed. Based on the COP displacement signals, the ApEn values were calculated. The results indicated that the prototype device was capable of capturing the increase in regularity of postural control in the visual-deprivation conditions. It was also able to decipher the subtle postural control differences along anterior–posterior and medial–lateral directions. The data analysis demonstrated that the prototype would enable the quantification of postural stability and thus provide a low-cost portable device to assess many conditions related to postural stability and human balance such as aging and pathologies.

## 1. Introduction

Measurement of the displacement of the center-of-pressure (COP) is an important tool used in biomechanics to assess postural stability. The ultimate goal of this research is to design and validate a portable low-cost device that can serve as an objective measure for quick assessment of postural stability and human balance related conditions. Approximate entropy (ApEn) is a good indicator of the irregularity of complex time series. The theoretical foundation of this study is that ApEn can be used as an effective indicator of subtle postural control impairments related to aging and pathologies [1]. For example, previous research suggests that younger subjects are generally more robust to perturbation than older subjects, in the sense that COP displacement increases with age in various conditions [2]. The ApEn values of COP oscillations would be lower to indicate the reduction of irregularity of the COP oscillations as people age. This provides additional information about age-related postural stability changes. Therefore, this device can potentially be used as a low-cost, portable, quick indicator of whether or not the subject suffers an increased risk of age-related falls [3].

Recently, Huang et al. [4] designed a force platform called center of pressure and complexity monitoring system (CPCMS) to measure the COP signals to quantify human balance using multivariate multiscale entropy. Sways tests were carried out using both the CPCMS and commercial Advanced Mechanical Technology Incorporation (AMTI) force plates to demonstrate that the CPCMS force platform can achieve similar results in terms of quantifying human balance. This research implements a similar approach in terms of hardware design to quantify human balance and postural stability by designing a low-cost portable device. However, the approach to validate the device, comparing with CPCMS, was significantly different. Instead of comparing with commercial force platform measurements, this study aimed to develop a postural control metric and compare those values with prior studies of postural control to find out if it is capable of successfully detecting subtle balance impairments.

Many researchers have studied balance impairments. Approximate entropy has been widely used as a biomechanical measure of postural stability [5,6,7,8,9,10,11]. Cavanaugh et al. used ApEn to detect subtle changes in COP oscillations when a secondary cognitive task was introduced [5]. Cavanaugh et al. [6] also used ApEn to assess changes in the randomness of COP oscillations after cerebral concussions. It was suggested that quiet standing with eyes open and closed on a stable platform may be the only sensory conditions that need to be assessed. Further research [7] revealed that ApEn values for COP time series provided important information related to the status of concussion. Overall, prior research [8,9,10] indicates that measures of postural stability would provide a useful clinical tool for return-to-play decisions in sport.

It is generally assumed that postural sway COP regularity differs with eyes open and closed while maintaining a quiet, upright stance. With eyes open, postural sway tends to be more random but less variable; whereas with eyes closed, postural sway tends to be less random but more variable [11]. Even though the visual system is the dominant sensory system for postural balance, some studies also indicated that vestibular [12,13] and somatosensory [13] disorders may significantly affect postural balance. Both visual and vestibular channels are responsible for compensatory action of balance control, however fundamental differences exist between them. The visual channel responds to the direction of a moving object, whereas the vestibular channel responds entirely to the motion of the head. When the visual channel is suppressed, the nervous system is able to re-weigh its available sensory systems in order to optimize stance control [14]. More research findings [15,16] indicate that the regularity of COP trajectories is clearly associated with the amount of attention devoted to postural control. Increased attention in various situations such as with eyes closed (vs. with eyes open) [15], or standing (vs. sitting) [16], tends to make the postural control less efficient, as evidenced by increased COP regularity. As a result, the postural control during quiet standing may provide instrumental information pertaining to aging, pathologies, athletic skill level [17] and cognitive manipulation [18]. The aim of the present study was to design and validate a low-cost, portable device to quantify postural stability so as to provide a useful tool for future postural control related research and applications.

## 2. Related Work

### 2.1. Center-of-Pressure (COP)

**Figure 1:**
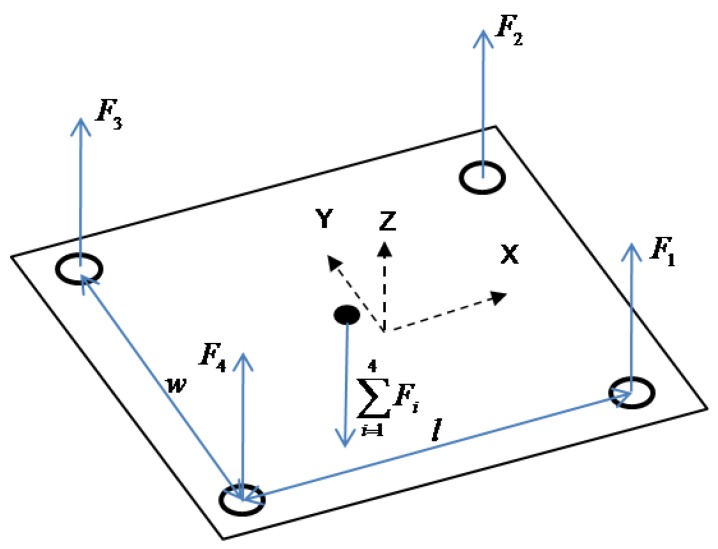
Center-of-pressure (COP) calculation in a 3D Cartesian coordinate system.

The COP is a simple parameter that consists of a time series of two coordinates in the X–Y plane. To calculate the COP, a typical 3D Cartesian coordinate system can be created at the centre of the top plate as shown in Figure 1, where X is the medial–lateral (ML) direction, Y is the anterior–posterior (AP) direction, and Z is the vertical direction.

Assuming that the readings of the four load cells at the four corners of the force platform are $F_{1}$, $F_{2}$, $F_{3}$, and $F_{4}$, then the moment about the Y axis can be represented as $M_{Y}=(F_{1}+F_{2}-F_{3}-F_{4})\cdot l/2$, which is equal to the product of COP X coordinate $X_{cop}$ and the summation of forces in the Z direction $F_{1}+F_{2}+F_{3}+F_{4}$ [4]. Therefore, the COP coordinates in the X and Y direction can be calculated using Equations (1) and (2), respectively.

(1) $$X_{cop}=\frac{(F_{1}+F_{2}-F_{3}-F_{4})\cdot l/2}{F_{1}+F_{2}+F_{3}+F_{4}}$$

(2) $$Y_{cop}=\frac{(F_{2}+F_{3}-F_{1}-F_{4})\cdot w/2}{F_{1}+F_{2}+F_{3}+F_{4}}$$

where l is the longitudinal distance between two load cells and w is the transverse distance between two load cells as indicated in Figure 1. The planar trajectory of the COP during balance control is called stabilogram, which is a scatter plot created by plotting simultaneous pairs of the COP locations in the ML and AP directions. It is an important technique to determine when an individual loses balance and to find measurable variables to quantify COP motions [19]. The shape of the stabilogram indicates the magnitude of postural sway in both ML and AP directions. Long and straight segments of the stabilogram represent sudden corrections, whereas short ones represent finer postural control. The COP motions usually differ between the eyes open and eyes closed conditions [20]. To quantify the stabilogram plots, different methods of quantifying COP variations such as velocity of COP displacement and critical time interval were developed by Raymakers et al. [20].

### 2.2. Approximate Entropy (ApEn)

ApEn was first proposed by Pincus [21] as a measure of changing system complexity based on the evaluation of time series data collected from the system. It has been increasingly used to discern levels of complexity in biological data sets. Pincus [21] demonstrated that ApEn is able to quantify complexity within relatively short data sets (as short as 75 to 100 data points). This is a great advantage for experiments that involve human subjects, particularly older or neurologically impaired subjects. For a given time series, ApEn is usually a real unitless number between 0 and 2. Theoretically, a perfectly random time series would yield an ApEn value close to 2, whereas a perfectly repeatable time series would yield an ApEn value close to 0. The ApEn value is larger if the time series is more complex and irregular. Mathematically, the ApEn formula we adopted is defined as,

(3) $$ApEn(N,m,r)=\frac{\sum \ln(\Phi^{m}(r))-\sum \ln(\Phi^{m+1}(r))}{N-m+1}$$

where, *N* = 300 is the total number of data points, *m* = 2 is the length of a data segment, and *r* = 0.2 multiplied by the standard deviation of the data points is the so-called tolerance threshold for accepting similar patterns between the neighbouring segments. In this study, a moving window of two elements will be applied to determine the probability that short sequences are repeated for multiple time series of 30 s.

Richman and Moorman [22] introduced sample entropy (SampEn) to counteract relatively inconsistent results of ApEn. Instead of counting each sequence as matching itself in ApEn algorithms, SampEn does not include self-matches in calculating the probability to maintain relative consistency. Although SampEn is less sensitive to data length, relatively more consistent and does not contain the inherent bias, short data sets would be deemed more difficult for SampEn due to the fact that in a small data set overlapping templates tend to inflate the variance of SampEn [23]. Since the key applications of this low-cost device in the future would most likely involve older or neurologically impaired test subjects, ApEn is chosen for this study.

For biological data sets, *m* = 2 and *r* = 0.2 were frequently chosen in literature, however it is widely known that the optimized choices of *m*, *r* and *N* are crucial for both ApEn and SampEn algorithms. Yentes et al. [24] carried out a detailed study on this and set out to test three hypotheses: (1) the value of ApEn and SampEn would change as a function of *m*, *r* and *N*; (2) SampEn demonstrates better consistency than ApEn and (3) both algorithms would be able to discriminate between theoretical and experimental data. Unfortunately, only the first hypothesis was fully supported by the results. Therefore, extreme caution should be exercised to choose the parameters for biological data. Among the three parameters: *m*, *r* and *N*, it is recommended that *N* should be greater than 200 and the length of the data should be sufficient to capture the biological complexity of the postural sway. Therefore, we believe that *N* = 300 (10 Hz for 30 s) may be the most appropriate for this study, considering the issues of the limited memory of a microcontroller and pathological populations that can fatigue easily. The parameter *m* = 2 is chosen for this study since it generally provides reasonable results in clinical data research [24]. Another advantage of choosing *m* = 2 is that it allows direct comparison to previously published results since *m* = 2 is frequently used in literature. It was also revealed that although typical value of *r* = 0.2 may not be the best all the time, there does not seem to be a better alternative [24]. We will carry out a study of *r* later using the data collected to verify that *r* = 0.2 is indeed appropriate for our biological data.

## 3. Materials and Methods

**Figure 2:**
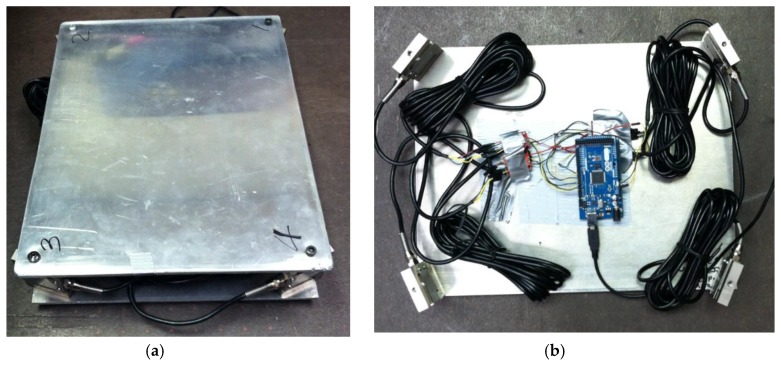
The portable postural stability testing device: (**a**) Overview of the assembled device; and (**b**) Detailed view of the data acquisition system.

Commonly used force platforms in biomechanics research, such as the ones manufactured by AMTI, cost more than ten thousand dollars. It is not a feasible approach to design a low-cost portable device using a commercial force platform [4]. To fabricate a low-cost force platform, two 6061 aluminum plates were used to create the basic structure. Four 100 Kg, S-type load cells (Product code RB-Phi-123 by Phidgets Inc., Calgary, AB, Canada) were placed at the four corners to connect the two plates as shown in Figure 2.

**Figure 3:**
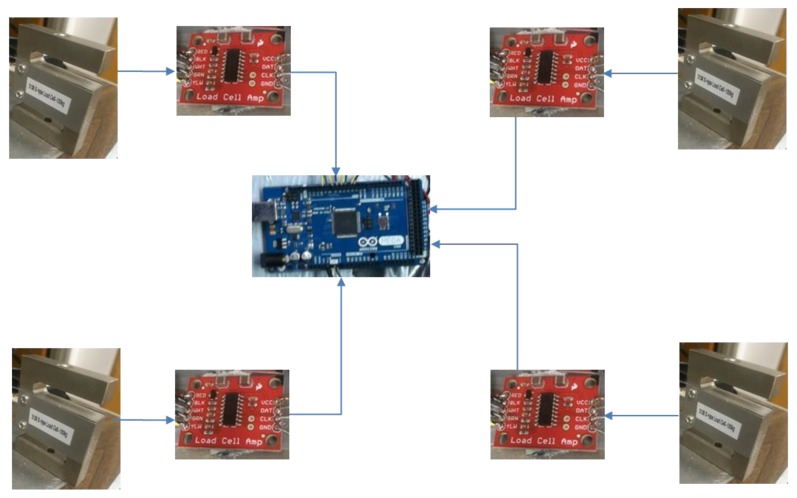
Schematic diagram of the load cells and amplifiers.

The amplifiers used to boost the signals from the load cells were HX711 SparkFun load cell amplifiers. An amount of 5 V from the Arduino microcontroller was used to power the amplifiers and load cells. A schematic diagram of the overall electrical system is shown in Figure 3.

**Figure 4:**
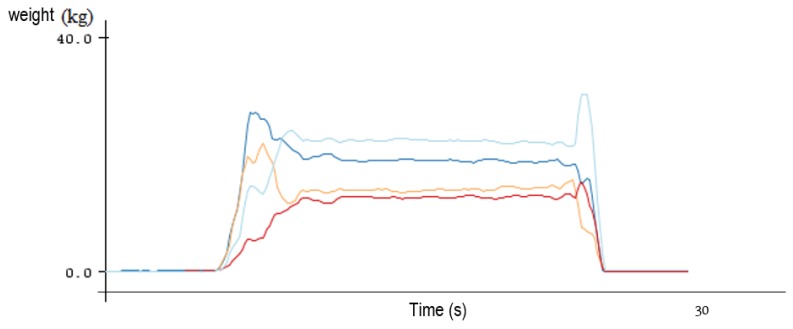
Plot of the raw load cell measures.

The measures of the four load cells are shown in Figure 4 while the test subject stepped on and subsequently stepped off the force platform. The signals were relatively clean, therefore no more additional filtering was needed. The signals were sampled at 10 Hz, which is a widely used sampling frequency according to previous studies [5,6,20] to reduce the influence of irrelevant noise in the data. There is little physiological significance above 10 Hz in the COP data [25]. The collected samples will be directly used to calculate the COP oscillations without down sampling [5,6].

**Figure 5:**
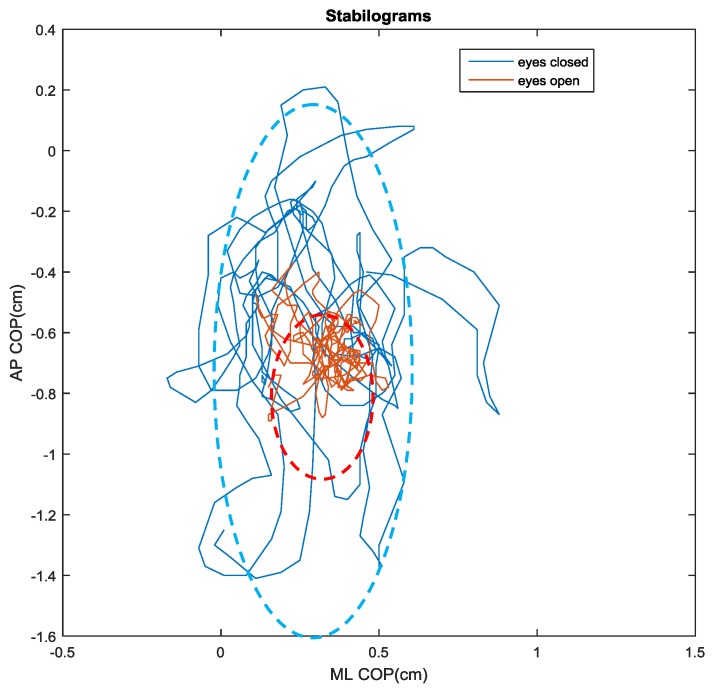
Stabilograms with/without visual feedback during quiet, upright standing (The horizontal axis is the medial–lateral (ML) direction and the vertical axis is the anterior–posterior (AP) direction).

Two exemplary stabilograms with/without visual feedback during quiet, upright standing are shown in Figure 5. It clearly demonstrates that the COP trajectory is more random but less variable with eyes open compared to eyes closed. The COP motion also demonstrates greater instability along the AP direction than the ML direction as indicated by the elliptical shapes in Figure 5.

**Figure 6:**
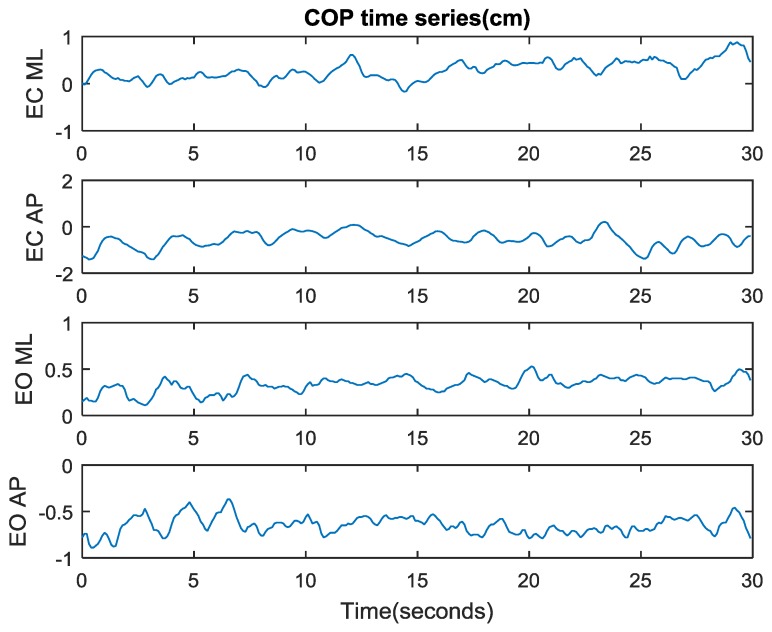
COP time series (represented by the vertical axis in cm) along the ML and AP directions with/without visual feedback during quiet, upright standing.

The same data is also shown in Figure 6 as the time series of COP trajectories along the ML and AP directions for eyes open (EO) and eyes closed (EC) conditions. It appears that the trajectories with eyes open demonstrate greater irregularity and smaller variability, suggesting an increase in the efficiency or automaticity of postural control. This is consistent with the previous study that shows that regularity of COP trajectories increases as the amount of attention invested in postural control increases (eyes being closed) [15,16].

The ApEn values for the four time series shown in Figure 6 are summarized in Table 1. It indicates that the COP time series in the AP direction with eyes open is most indeterministic, whereas the COP trajectory in the AP direction with eyes closed is most deterministic as demonstrated in Figure 6.

**Figure 7:**
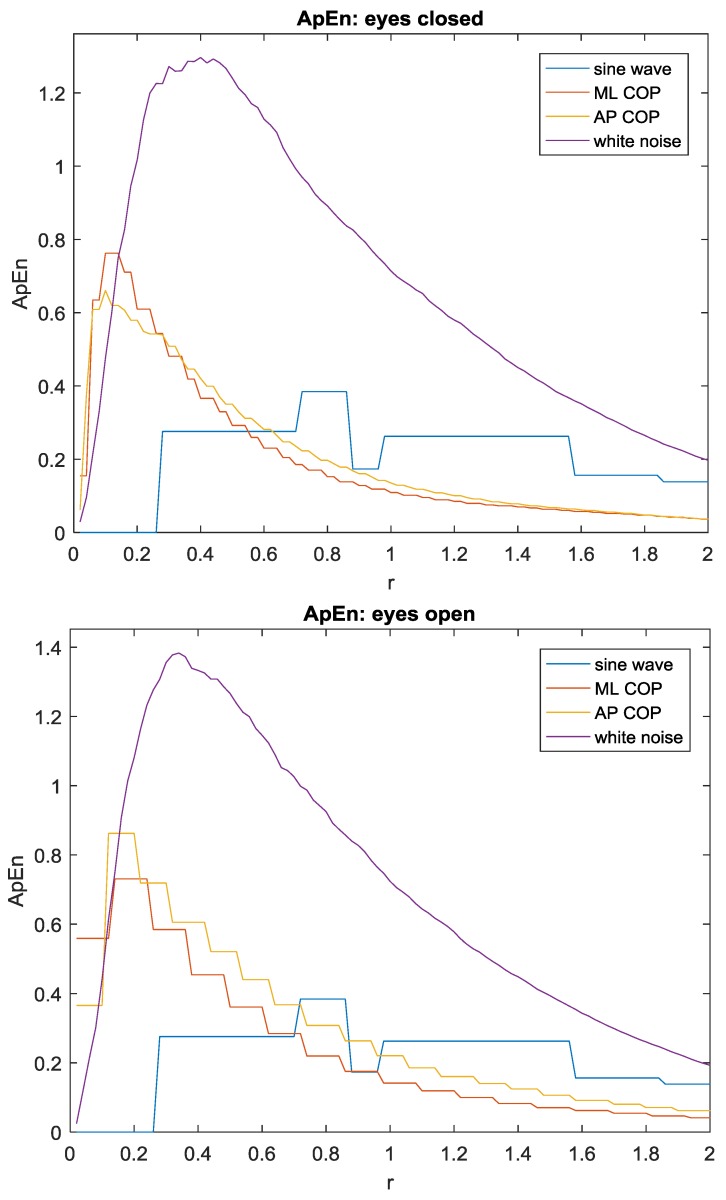
ApEn values of sinusoidal wave, ML COP trajectory, AP COP trajectory and white noise with various unitless thresholds (*r*) are shown for both eyes closed and eyes open conditions.

The selection of *r* is critical for the ApEn algorithm. Chon et al. [26] recently suggested that the widely recommended value for *r* can lead to an incorrect assessment. Instead, it was suggested that maximum ApEn leads to better prediction of a signal’s complexity. We carried out a study by choosing r between 0.02 and 2 with a step size of 0.02 to verify if the maximum ApEn occurs in the neighborhood of *r* = 0.2 in our data. As shown in Figure 7, choosing *r* = 0.2 appears to predict the maximum value of ApEn consistently for our biological data in both ML and AP directions. Since evaluating many different choices of *r* is a very cumbersome and time-consuming process [26], the maximum ApEn results shown in Figure 7 were evaulated offline using the collected experimental data (Figure 5). Real-time evaluation of multiple choices of *r* is not a computationally feasible approach for the low-cost portable device that we designed, therefore the ApEn algorithm with a carefully-chosen, fixed value of *r* = 0.2 was implemented in the present study.

## 4. Results

To retain a direct comparison of postural control with and without visual feedback, only one test subject was included in the present study. After the efficacy of this device is validated, more test subjects with various conditions such as aging or pathologies will be included in the future studies. A total of 30 pairs of data were collected for eyes closed and eyes open conditions. After a brief initialization process, the test subject would step on the force platform and maintain an upright, quiet stance for 60 s. The 30 s data with eyes closed was recorded by the microcontroller immediately after the 30 s data with eyes open was collected. The postures during the eyes open and eyes closed period were kept identical to the best of the test subject’s capability. The 30 pairs of data were randomly collected to guarantee that the data can represent the general trend that the device predicts. It is generally known that the removal of visual feedback increases the regularity of COP trajectories [15]. The parameters of the stabilogram (Figure 5) and the time series (Figure 6) during quiet, upright standing for both eyes closed and eyes open conditions are summarized in Table 2.

The ApEn values of the COP time series during the quiet, upright standing for both eyes closed (*n* = 30) and eyes open (*n* = 30) conditions are summarized in Table 3.

**Figure 8:**
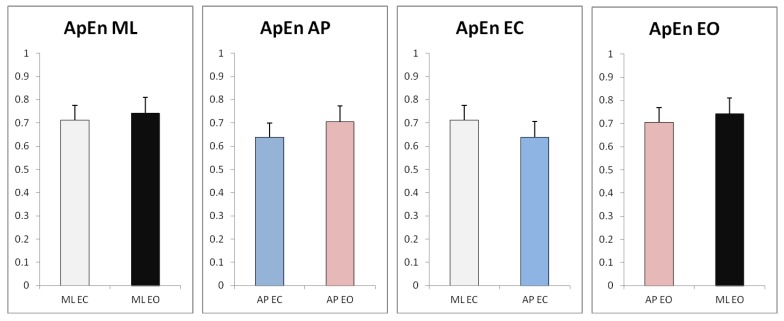
Comparison of ApEn values for quiet standing with and without visual feedback.

The results are also shown in Figure 8 to directly compare the different factors, i.e., eyes closed (EC) vs. eyes open (EO) and AP vs. ML direction.

## 5. Discussion

The observations of the stabilogram and the time series indicate that the postural stability in the AP direction (range = 1.62 cm) and the ML direction (range = 1.05 cm) with eyes closed is considerably less efficient than the postural stability in the AP direction (range = 0.52 cm) and ML direction (range = 0.42 cm) with eyes open. It is also clearly indicated that greater instability along the AP direction than the ML direction during quiet standing (1.62 > 1.05 and 0.52 > 0.42). This is consistent with literature and it is commonly understood that the passive mechanical constraints at ankle and knee joints largely minimizes the ML motion, while more degrees of freedom are available for the movements along the AP direction [27].

Collectively, the present study implies that standing with eyes open increases COP irregularity (higher ApEn values), indicating that postural control in both the AP and ML directions is relatively more efficient and automatic with visual feedback during upright, quiet standing. This result is consistent with the existing literature [11,15,28]. The postural control in the AP direction with eyes closed is much less efficient. Although the postural control variation in the ML direction between eyes closed and eyes open conditions did not demonstrate considerable difference, the mean value (*n* = 30) for eyes open (0.742) is still greater than the mean value for eyes closed (0.712), indicating that the postural control with eyes closed in the ML direction is still generally more deterministic. The interpretation as to why the variation in the ML direction is not as noteworthy may be due to the fact that postural control in the ML direction tends to be more stationary than AP counterparts in a normal postural control system [28]. It could also be due to the randomness associated with the amount of attention invested in postural control [15]. The attention factor, important though it is, cannot be explicitly regulated in the present study, which may skew the data one way or another. Furthermore, postural control is a rather complex skill that involves six important underlying physiological systems: control of dynamics, cognitive processing, biomechanical constraints, movement strategies, sensory strategies and orientation in space [29]. Disorder in any of the six sources may affect postural stability. One of the important resources involved in this study is cognitive processing. The more difficult the task, the more cognitive processing is invested. Thus, the performance in postural control becomes less efficient as the difficulty of the task increases (deprivation of visual feedback).

The results also imply that postural sway in the AP direction is generally more deterministic (lower ApEn values), meaning that it is more repeatable or more structured than the ML direction with or without visual feedback during upright, quiet standing. This result is again consistent with the existing literature [30]. The difference is more noteworthy with eyes closed. This implies that postural control in the ML direction is of greater importance in the visual-deprivation condition. This is consistent with the fact that the ML direction is generally more affected by aging or pathologies and the decline of postural stability in the ML direction can lead to increased risk of falling [31]. For the same reason, the decline of postural stability in the ML direction is less affected in the present study compared to the AP direction with visual deprivation, suggesting that the test subject only demonstrated slightly increased risk of falling in the visual-deprivation condition.

Taken together, current findings through this study show that our prototype device is capable of capturing the increase in regularity of postural sway with reduced visual feedback. We were also able to decipher how postural control changes with or without visual feedback and the distinction between the AP and ML directions. The ApEn index developed appears to be a reliable indicator for quantifying postural stability and detecting subtle abnormality related to aging or pathologies.

## Figures and Tables

**Table 1:** Approximate entropy (ApEn) values for quiet standing with and without visual feedback.

Direction	Eyes	ApEn
ML	Closed	0.64
AP	Closed	0.55
ML	Open	0.71
AP	Open	0.80

**Table 2:** Comparison of stabilogram parameters for quiet standing with and without visual feedback.

Direction	Eyes	Maximum (cm)	Minimum (cm)	Range (cm)
ML	Open	0.53	0.11	0.42
AP	Open	−0.37	−0.89	0.52
ML	Closed	0.88	−0.17	1.05
AP	Closed	0.21	−1.41	1.62

**Table 3:** Comparison of ApEn values for quiet standing with and without visual feedback.

Direction	Eyes	*n*	Mean	Standard Deviation
ML	Open	30	0.742	0.068
AP	Open	30	0.705	0.075
ML	Closed	30	0.712	0.063
AP	Closed	30	0.637	0.054

## References

[1] Buckley T.A., Oldham J.R., Caccese J.B. (2016). Postural control deficits identify post-concussion neurological deficits. J. Sport Health Sci..

[2] Abrahamova D., Hlavacka F. (2008). Age-related changes of human balance during quiet stance. Physiol. Res..

[3] Strang A.J., DiDomenico A.T. (2010). Postural control: Age-related changes in working-age men. Prof. Safety.

[4] Huang C., Sue P., Abbod M.F., Jiang B.C., Shieh J. (2013). Measuring center of pressure signals to quantify human balance using multivariate multiscale entropy by designing a force platform. Sensors.

[5] Cavanaugh J.T., Mercer V.S., Stergiou N. (2007). Approximate entropy detects the effect of a secondary cognitive task on postural control in healthy young adults: A methodological report. J. NeuroEng. Rehabil..

[6] Cavanaugh J.T., Guskiewicz K.M., Giuliani C., Marshall S., Mercer V., Stergiou N. (2005). Detecting altered postural control after cerebral concussion in athletes with normal postural stability. Br. J. Sports Med..

[7] Cavanaugh J.T., Guskiewicz K.M., Giuliani C., Marshall S., Mercer V.S., Stergiou N. (2006). Recovery of postural control after cerebral concussion: New insights using approximate entropy. J. Athl. Train..

[8] Guskiewicz K.M., Riemann B., Perrin D., Nashner L. (1997). Alternative approaches to the assessment of mild head injury in athletes. Med. Sci. Sports Exerc..

[9] Guskiewicz K.M. (2011). Balance assessment in the management of sport-related concussion. Clin. Sports Med..

[10] Riemann B.L., Guskiewicz K.M. (2000). Effects of mild head injury on postural stability as measured through clinical balance testing. J. Athl. Train..

[11] Bartlett R., Gratton C., Rolf C.G. (2010). Encyclopedia of International Sports Studies.

[12] García R.B., Corresa S.P., Bertomeu J.M.B., Suárez-Varela M.M.M. (2012). Static posturography with dynamic tests. Usefulness of biomechanical parameters in assessing vestibular patients. Acta Otorrinolaringol. Espanola.

[13] Gaerlan M.G. (2010). The Role of Visual, Vestibular, and Somatosensory Systems in Postural Balance. Master’s Thesis.

[14] Jáuregui-Renaud K. (2013). Postural Balance and Peripheral Neuropathy.

[15] Donker S.F., Roerdink M., Greven A.J., Beek P.J. (2007). Regularity of center-of-pressure trajectories depends on the amount of attention invested in postural control. Exp. Brain Res..

[16] Roerdink M., Hlavackova P., Vuillerme N. (2011). Center-of-pressure regularity as a marker for attentional investment in postural control: A comparison between sitting and standing postures. Hum. Mov. Sci..

[17] Lamoth C.J.C., van Lummel R.C., Beek P.J. (2009). Athletic skill level is reflected in body sway: A test case for accelometry in combination with stochastic dynamics. Gait Posture.

[18] Stins J.F., Roerdink M., Beek P.J. (2011). To freeze or not to freeze? Affective and cognitive perturbations have markedly different effects on postural control. Hum. Mov. Sci..

[19] Hasan S.S., Lichtenstein M.J., Shiavi R.G. (1990). Effect of loss of balance on biomechanics platform measures of sway: Influence of stance and a method for adjustment. J. Biomech..

[20] Raymakers J.A., Samson M.M., Verhaar H.J.J. (2005). The assessment of body sway and the choice of the stability parameter(s). Gait Posture.

[21] Pincus S.M. (1991). Approximate entropy as a measure of system complexity. Proc. Natl. Acad. Sci. USA.

[22] Richman J.S., Moorman J.R. (2000). Physiological time-series analysis using approximate entropy and sample entropy. Am. J. Phys. Heart Circ. Phys..

[23] Johnson M.L., Brand L. (2004). Numerical Computer Methods.

[24] Yentes J.M., Hunt N., Schmid K.K., Kaipust J.P., McGrath D., Stergiou N. (2013). The appropriate use of approximate entropy and sample entropy with short data sets. Ann. Biomed. Eng..

[25] Borg F.G., Laxaback G. (2010). Entropy of balance—Some recent results. J. NeuroEng. Rehabil..

[26] Chon K.H., Scully C.G., Lu S. (2009). Approximate entropy for all signals: Is the recommended threshold value R appropriate?. IEEE Eng. Med. Biol. Mag..

[27] Valle M.S., Casabona A., Fiumara A., Castiglione D., Sorge G., Cioni M. (2016). Quantitative analysis of upright standing in adult with late-onset Pompe disease. Sci. Rep..

[28] Ghomashchi H., Esteki A., Nasrabadi A.M., Sprott J.C., BahrPeymaInt F. (2011). Dynamic patterns of postural fluctuations during quiet standing: A recurrence quantification approach. J. Bifurc. Chaos.

[29] Horak F.B. (2006). Postural orientation and equilibrium: What do we need to know about neural control of balance to prevent falls?. Age Ageing.

[30] Balasubramaniam R., Riley M.A., Turvey M.T. (2000). Specificity of postural sway to the demands of a precision task. Gait Posture.

[31] Eduardo L., Lizama C., Pijnappels M., Peter Reeves N., Verschueren S.M.P., van Dieën J.H. (2016). Can explicit visual feedback of postural sway efface the effects of sensory manipulations on mediolateral balance performance?. J. Neurophysiol..

